# Do All Coccidia Follow the Same Trafficking Rules?

**DOI:** 10.3390/life11090909

**Published:** 2021-08-31

**Authors:** Virginia Marugan-Hernandez, Gonzalo Sanchez-Arsuaga, Sue Vaughan, Alana Burrell, Fiona M. Tomley

**Affiliations:** 1 The Royal Veterinary College, University of London, Hawkshead Lane, North Mymms AL9 7TA, UK; gsanchezarsuaga@rvc.ac.uk (G.S.-A.); ftomley@rvc.ac.uk (F.M.T.); 2 Department of Biological and Medical Sciences, Oxford Brookes University, Gipsy Lane, Oxford OX3 0BP, UK; svaughan@brookes.ac.uk; 3 Electron Microscopy Science Technology Platform, The Francis Crick Institute, London NW1 1AT, UK; alana.burrell@crick.ac.uk

**Keywords:** Coccidia, *Eimeria* species, *Toxoplasma gondii*, protein trafficking, apical complex, endogenous development

## Abstract

The Coccidia are a subclass of the Apicomplexa and include several genera of protozoan parasites that cause important diseases in humans and animals, with *Toxoplasma gondii* becoming the ‘model organism’ for research into the coccidian molecular and cellular processes. The amenability to the cultivation of *T. gondii* tachyzoites and the wide availability of molecular tools for this parasite have revealed many mechanisms related to their cellular trafficking and roles of parasite secretory organelles, which are critical in parasite-host interaction. Nevertheless, the extrapolation of the *T. gondii* mechanisms described in tachyzoites to other coccidian parasites should be done carefully. In this review, we considered published data from *Eimeria* parasites, a coccidian genus comprising thousands of species whose infections have important consequences in livestock and poultry. These studies suggest that the Coccidia possess both shared and diversified mechanisms of protein trafficking and secretion potentially linked to their lifecycles. Whereas trafficking and secretion appear to be well conversed prior to and during host-cell invasion, important differences emerge once endogenous development commences. Therefore, further studies to validate the mechanisms described in *T. gondii* tachyzoites should be performed across a broader range of coccidians (including *T. gondii* sporozoites). In addition, further genus-specific research regarding important disease-causing Coccidia is needed to unveil the individual molecular mechanisms of pathogenesis related to their specific lifecycles and hosts.

## 1. Introduction

Apicomplexan parasites possess a complex endomembrane system, which includes specialized secretory organelles critical for parasite invasion and the formation of a parasitophorous vacuole (PV), the intracellular compartment within which the parasite resides and interacts with the host cell [1]. Previous studies in Apicomplexan parasites, such as *Toxoplasma gondii* and *Plasmodium* species, have established that apicomplexans have repurposed the exocytic and endocytic pathways of higher eukaryotes to evolve their specialized regulatory secretory organelles [2,3,4]. This rearrangement has led apicomplexans to possess a reduced endomembrane system compared to higher eukaryotes, which is efficient enough to perform the protein trafficking, targeting, processing and recycling needed for their obligate intracellular lifestyles.

*Eimeria* species are apicomplexan parasites closely related to *T. gondii* and other members of the subclass Coccidia (*Cystoisospora* spp., *Isospora* spp., *Neospora caninum*, *Sarcocystis* spp.). There are no species of *Eimeria* that infect humans. However, members of the genus, which comprises > 1800 named species to date, can potentially infect the majority of vertebrates, causing coccidiosis [5], a disease with a significant impact on the rearing of livestock and poultry worldwide. Recent studies have estimated that coccidiosis costs > £10 billion per annum to the global poultry sector [6], making coccidiosis a target for active research in academia and industry. Whereas work on *T. gondii* provides valuable insights into the mechanisms of coccidian invasion, differences in the specific infection biology between parasites, such as host-range, tissue- and site-specificity, PV formation, the mechanism of asexual cellular division, and pathological interaction with the host, suggests that it is not always possible to directly infer functions in *Eimeria* species from the information gathered in *T. gondii* [7].

## 2. Coccidian Lifecycles and Endogenous Development

*Eimeria* parasites exhibit an acute monoxenous and strictly host-specific lifecycle in contrast to the heteroxenous *T. gondii*, which displays both acute and chronic phases and can infect a wide range of intermediate hosts (including humans). *Eimeria* endogenous development is tightly regulated and self-limiting. Ingested oocysts release sporozoites that infect cells of the intestinal epithelium (tissue-specific), where they undergo a fixed number of rounds of asexual replication (schizogony), releasing merozoites. This process is followed by sexual reproduction (gametogony) to form new oocysts. Then, these new oocysts are voided to the environment via the feces, where sporulation (sporogony) takes place [7]. Similarly, when ingested by felids (definitive host), *Toxoplasma* oocysts (or tissue cysts) release sporozoites (or bradyzoites) that, after the invasion of host cells, undergo asexual (endopolygeny) and sexual (gametogony) replication in the gut epithelium, producing oocysts that are released to the environment [8]. However, when *Toxoplasma* infects any of its many intermediate hosts, parasites transform into tachyzoites in the gut. These tachyzoites transiently infect many tissues before they migrate to the neural and muscle tissues [9]. Tachyzoites undergo an indefinite number of rounds of asexual cell division until the pressure of the immune system leads to the conversion intobradyzoites within the tissue cysts, where they remain for life [10]. It is important to highlight that most investigations into *T. gondii* protein trafficking and secretion have focused on the tachyzoite stage [4].

**Figure 1:**
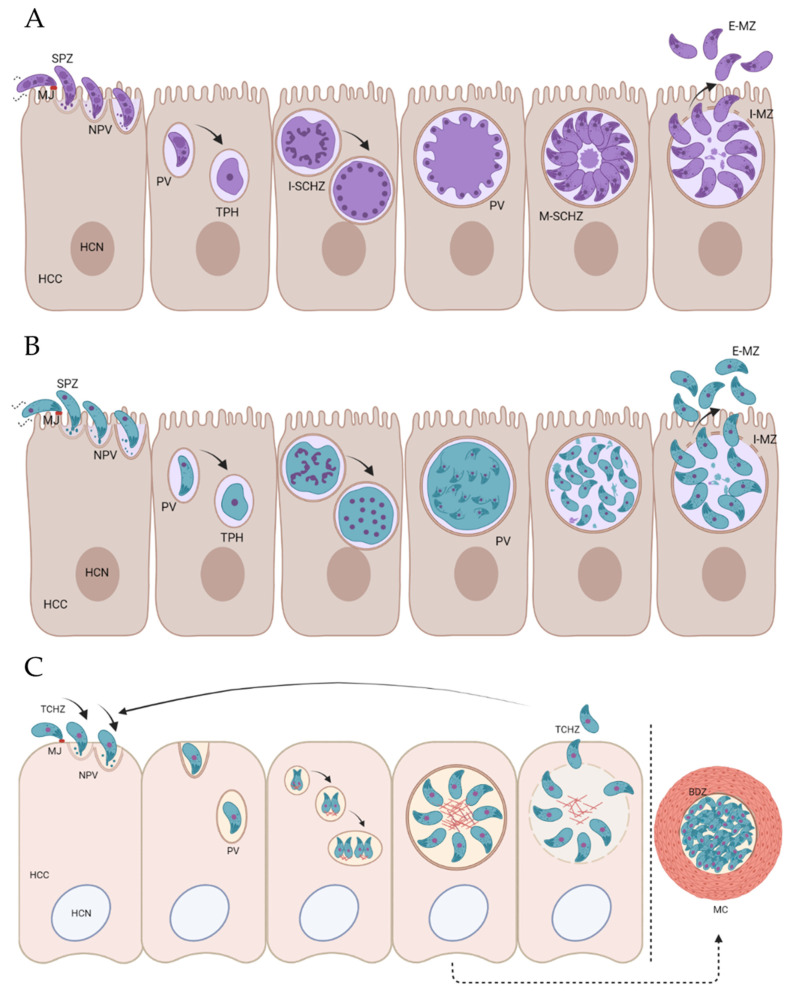
Invasion and endogenous development of *Eimeria* spp. sporozoites (**A**), *T. gondii* sporozoites (**B**), and *T. gondii* tachyzoites (**C**). (**A**) Schizogony: *Eimeria* sporozoites (SPZ) attach to the surface of enterocytes [17], creating the moving junction (MJ; [18]), a specialized structure between the parasite and the host cell. Invasion continues with the invagination of the host cell membrane, leading to the creation of the nascent parasitophorous vacuole (NPV). This internalization ends with the sealing of the membrane at the site of the parasite entry, conforming the parasitophorous vacuole (PV), in which the parasite resides and continues its endogenous development. Sporozoites develop inside the PV into rounded and growing trophozoites (TPH). As the parasite grows, the trophozoite undergoes multiple nuclear divisions to produce a large multinuclear cell. In the immature schizont (I-SCHZ), the nuclei of the parasite stage have a random distribution, migrating to the periphery (close to the parasite plasma membrane) to accommodate merozoite development [13]. Merozoites start budding around each nucleus and grow radially inside the PV. At the end of this phase, the division of the cytoplasm results in the formation of mononuclear spindle-shaped, motile daughter cells (intracellular merozoites (I-MZ)), which reside inside the mature schizont (M-SCHZ) until release from the cell. Merozoites escape into the intestinal lumen, where extracellular merozoites (E-MZ) invade new cells and restart the cycle (or develop to the sexual stages). (B) Endopolygeny: *T. gondii* sporozoites attach to the surface of enterocytes in the definitive host and form the MJ. Invasion continues with the invagination of the host cell membrane, forming the NPV and the PV once sporozoite internalization is completed. Sporozoites develop inside the PV into a TPH, which undergoes multiple rounds of DNA replication and mitosis without the division of the cytoplasm to produce a large multinuclear cell. Then, multiple daughter cells develop by internal budding within the cytoplasm [14]. Residual cytoplasm disappears as merozoites mature (I-MZ) and are released from the host cell (E-MZ) to enter new cells and repeat the cycle (or develop to the sexual stages). (C) Endodyogeny: The internalization of *T. gondii* tachyzoites (TCHZ) commences with the formation of the MJ. Once inside the host cell (HCC), tachyzoites develop rapidly inside the PV, where single rounds of DNA replication lead to the formation of two daughters within the mother cell cytoplasm prior to the completion of nuclear division. As the parasite replicates, new daughter cells arrange radially to form the mature PV, whose rupture results in the release of tachyzoites that could indefinitely repeat this lytic cycle. Eventually, under the pressure of the immune system, tachyzoites can differentiate into slowly dividing bradyzoites (BDZ) within the PV, leading to the formation of the ‘tissue cyst’ [15]. Enclosed in a thick cyst wall formed from an infolding of the host cell membrane, these bradyzoites can persist for the lifetime of the host, usually in cells of the brain and/or skeletal muscles (MC). HCN: Host cell nucleus. Figure created with BioRender.com.

Stage differentiation in coccidian parasites involves significant changes at the transcriptional and cellular levels [11]. The endogenous development for *Eimeria* sporozoites, *T. gondii* sporozoites and *T. gondii* tachyzoites is detailed in Figure 1. All coccidian zoites have a conserved apical complex that is used for the active invasion of the host cells [12]. Changes at the cellular level have been described in the *Eimeria* species during the development of sporozoites to schizonts. For example, in cell culture at 24 h post-invasion, most intracellular structures of *E. tenella* sporozoites were retained, whereas by 35 h, most structures had disappeared, and only the nucleus, mitochondria, endoplasmic reticulum, and refractile bodies were identifiable [13]. *Eimeria* sporozoites (and merozoites) undergo schizogony (Figure 1A), which involves several rounds of DNA replication and nuclear division, followed by the simultaneous budding of daughters at the plasma membrane and eventual release [13]. In a similar manner, *T. gondii* sporozoites and merozoites divide by endopolygeny, with DNA replication, mitosis, and daughter cell budding all taking place internally within the cytoplasm (Figure 1B) [14]. In contrast, *T. gondii* tachyzoites divide by endodyogeny (Figure 1C), which involves several single rounds of DNA replication and nuclear division, followed by internal budding to form pairs of daughters (daughters form in the cytoplasm, not at the plasma membrane) [15]. PV-containing *T. gondii* tachyzoites support longer durations of parasitism compared to *Eimeria* schizonts, lasting throughout chronic infection, which involves the conversion of tachyzoites into slow-growing bradyzoites within the PV and the formation of the tissue cyst (Figure 1C). Both tachyzoites and bradyzoites of *T. gondii* conserve many cellular structures that are typical of coccidian cells [16]. Nevertheless, significant differences have been reported between these two stages, such as a posterior location of the nucleus in bradyzoites, as well as higher content of amylopectin granules [15].

We hypothesize that the similarities in the zoite invasion mechanisms, differences in endogenous development, and the acute versus chronic lifecycles of *Eimeria* sporozoites and *T. gondii* tachyzoites described above (Figure 1) may directly correlate to the mechanisms of protein trafficking and secretion that each parasite has evolved. In this manuscript, we detail the main similarities and differences found in the literature linking protein trafficking and secretion with the mechanisms of invasion and development.

## 3. Coccidians Share Mechanisms for the Trafficking and Secretion of Proteins to the Cell Surface, Micronemes and Rhoptries

### 3.1. Early Host-Parasite Interactions and Main Proteins Involved

The initial interaction of coccidian protozoa with host cells involves surface molecules from both the parasite and host cells [19,20]. Proteins expressed on the surface of the parasite have important roles in the recognition, adhesion, and invasion of the host cells, as well as in evasion of the host immune responses [21]. Superfamilies of glycophosphatidylinositol (GPI)-linked surface antigens, termed surface antigens (SAGs), have been widely studied in coccidians, including *Eimeria* [22,23]. Interestingly, Ramly and collaborators [24] recently reported that the *Eimeria* SAG family, which has a low level of sequence similarity across its >80 members, is unified by a 3-layer αβα fold, which acts as a platform to present the distal surface areas of hypervariability to the host cell in a manner similar to that reported for the unrelated tandem β sandwich domain structure of the *T. gondii* SRS (SAG-related sequences) family [25]. GPI-anchors at the C-terminal of SAG proteins appear to contain all the targeting signals required for this external localization, as specific sequences from TgSAG1 are capable of relocating bacterial alkaline phosphatase [26] and TgMIC6 to the *T. gondii* tachyzoite surface [27]. In *E. tenella*, the addition of the GPI-anchor of EtSAG1 also relocates the fluorescent reporter mCherry onto the surface of sporozoites [28], evidencing that it likely also contains the necessary elements for surface localization. Nevertheless, the specific vesicles transporting GPI-anchored proteins and the trafficking pathway followed to reach the surface have not yet been elucidated. It is of note, however, that not all GPI-anchored proteins traffic to the surface. In particular, TgSUB1, which is GPI-anchored, is stored in micronemes [29].

After this initial interaction, the apical complex plays a critical role in the parasite adhesion and active invasion of the host cell. The apical complex is a defining feature of apicomplexan protozoan parasites, after which the phylum is named. This prominent structure was described over 40 years ago in *T. gondii* [30]. The apical complex is composed of an apical polar ring to which a set of helically arrayed subpellicular microtubules are attached [31], including the conoid, an additional apical cone-like structure composed of tightly wound tubulin fibers associated with two pre-conoidal rings and a pair of intraconoidal microtubules; and the apical organelles, micronemes, and rhoptries, which are part of the parasite endomembrane system. *Eimeria* micronemes and rhoptries are generated de novo during the formation of sporozoites [32] and merozoites [33].

Micronemes secrete adhesive proteins (MICs), which form adhesin complexes in a timely fashion upon contact with the host cell [17,27,34,35]. They span the parasite plasma membrane, and this surface location is critical for parasite attachment to the host cells, gliding, the migration across tissues, and the formation of a moving junction (MJ) between the parasite and host membranes in cooperation with the RON proteins secreted from the neck of the rhoptries [18] and observed in the sporozoites of *E. tenella* [36]. The MJ mediates host cell invasion and the formation of the PV, acting as a molecular sieve of the host proteins to prevent endosome fusion and lysosomal destruction [37]. In addition to the RONs, rhoptries secrete ROPs from the bulb portion, which play a role in cell manipulation (kinases). These ROPs have also been reported as important virulence factors in *T. gondii* [38,39]. There is high conservation between the *Eimeria* and *T. gondii* MIC [40] and RON proteins involved in the first stages of endogenous development [41], whereas there is a much more limited conservation of the ROP proteins [41,42]. A specific clade of seven ROP kinases subfamilies, including both predicted active and pseudokinases, has been described for *E. tenella* [43].

### 3.2. Essential Molecules and Signals for Trafficking to Micronemes and Rhoptries

In Coccidia, the MIC and RON/ROP proteins traffic through the endoplasmic reticulum (ER), Golgi, and trans-Golgi network (TGN), and are then sorted to endosome-like compartments (ELC) bearing early (Rab5) or late (Rab7) endosome markers. An essential step in organelle formation is the proteolytic maturation of the MICs and RON/ROPs [44], which most likely occurs within or during exit from the ELC [45] and is mediated by proteinases, including aspartyl protease 3 [46]. The mechanisms for this trafficking pathway described in *T. gondii* are likely to be conserved in the *Eimeria* species, since the gene orthologues to essential molecules for TGN to ELC trafficking are present in different *Eimeria* genomes (Table 1; www.toxodb.org (accessed on 26 August 2021)). Divergence between MIC and RON/ROP proteins at the ELC has been noted. Immature MICs (TgM2AP and TgMIC5) located in the ELC have contain endocytosed host proteins, while immature RONs (TgRON4) in the ELC lack ingested host proteins [45].

The signals governing intracellular trafficking to the secretory organelles are not fully understood. However, there are a number of studies in *T. gondii* that have described specific N-terminal pro-peptide domains for targeting proteins for storage in the micronemes or rhoptries before their release during invasion (e.g., TgM2AP [57], TgMIC3 [58], TgMIC5 [59], TgROP1 with at least two rhoptry targeting signals [60], TgSUB1 [61]). In addition, trafficking dependent on tyrosine residues in the cytoplasmic tails of transmembrane of TgMIC2 and TgROP2 has been reported [62,63], suggesting the conservation of tyrosine-dependent sorting found in higher eukaryotes [64]. Interestingly, Gaji and collaborators [65] proved that pro-peptide domains are not only interchangeable between different *T. gondii* MICs, but also with *E. tenella* EtMIC5, where the EtMIC5 pro-peptide can efficiently deliver TgM2AP to the micronemes. Hyunh and collaborators [66] also demonstrated that *E. tenella* EtMIC1 localizes in the micronemes when expressed in *T. gondii* and that this protein can partially compensate the role of its orthologous TgMIC2. More recently, the expression of *E. tenella* EtROP1 in *T. gondii* sporozoites showed colocalization with TgROP2, evidencing the presence of equivalent targeting signals in both parasite species [42]. These studies strongly suggest a common mechanism of trafficking for apical secretory organelles within the Coccidia.

The existence of more than one trafficking pathway to micronemes, leading to either distinct populations containing different MICs or a single population with different sub-compartments, has been also proposed for *T. gondii* [67]. Previous studies in *E. tenella* have shown a lack of colocalization of some EtMIC proteins (e.g., EtMIC3/EtAMA1 and EtMIC3/EtMIC5 in sporozoites [36], and EtMIC2/EtAMA2 in second-generation merozoites [68]), which could align with this hypothesis of zoites containing distinct populations of micronemes. Nonetheless, it cannot be ruled out that this could reflect differences in the timing and levels of individual MIC protein expression.

### 3.3. Regulation of Protein Secretion from Micronemes and Rhoptries

During invasion, the coordinated exocytosis of micronemes and rhoptries isessential [69]. Recent studies using spatial proteomics and high-resolution microscopy have identified new conoid proteins, demonstrating that this is a conserved apicomplexan structure, albeit in varying forms [12]. Analysis of the apical complex of *T. gondii* has allowed a detailed characterization of how the intraconoidal microtubules and associated secretory vesicles are involved in the microneme and rhoptry spatial organization around the conoid [70].

MIC secretion occurs from the apical pole when coccidian parasites contact host cells [17,71]. Contact activates a parasite cGMP signaling pathway with two major effectors: (1) Inositol 1,4,5-triphosphate (IP_3_) stimulates intracellular calcium release, activating calcium-dependent protein kinases (CDPKs). Phosphorylate proteins are involved in microneme exocytosis and the activation of the parasite glideosome [72]. (2) Diacyl-glycerol (DAG) is converted to phosphatic acid at the inner leaflet of the parasite plasma membrane, which is sensed by pleckstrin-homology domain containing protein (APH) located on the surface of apical micronemes [73]. Despite these advances in knowledge, the precise mechanism of microneme exocytosis remains enigmatic. Current models speculate that for the coccidia, apical micronemes are channeled through the conoid and fuse at the tip of the parasite [74].

Rhoptry proteins can access the host cytosol, and some can reach the host cell nucleus, affecting signaling pathways [75]. As for MICs, RONs, and ROPs, secretion occurs at the apical pole, requiring previous MIC discharge [76]. However, the way that ROPs cross both the parasite and host membranes remains unknown. Recent advances have defined further molecules and structures involved in rhoptry discharge, such as the orthologues of *Paramecium* non-discharge rosette complexes [77]; rhoptry apical surface proteins (RASPs), which cap the extremity of the rhoptries and play a role in exocytosis [78]; and the calcium-sensing ferlins involved in rhoptry secretion [79]. Interestingly, when host cell invasion is inhibited, the parasites attach, form a tight junction with the host plasma membrane, and discharge rhoptry contents such that nascent parasitophorous-empty vacuoles are observed [80]. The existence of a pore that connects the rhoptry to the host cytosol was hypothesized in 1983 by Nichols and collaborators [81] following their observation of a rhoptry opening in a thin section of the electron microscopy of *T. gondii*. In 2012, this pore was suggested again, but not fully resolved, by Paredes-Santos and collaborators [70], who used focused ion beam scanning electron microscopy to describe the structural elements linked to rhoptry secretion. This was discussed again in 2021 by Sparvoli and collaborators in a recent review, discussing the limitations of studying rhoptry secretion due to the lack of inducers and the visualization an opening to the host cell (on several occasions) without a final confirmation whether the opening represents an open or transient pore. Nevertheless, rhoptry exportation through the pore has not been demonstrated so far [82]. Although the presence of ROPs in the host cytosol has not been directly observed in the *Eimeria* species, Diallo and collaborators [42] proved the interaction of EtROP1 with the host tumor protein p53, leading to the inhibition of host cell apoptosis and G0/G1 cell cycle arrest, supporting the idea of rhoptries reaching the host cytosol/nucleus.

## 4. Coccidian Stages Diverge in Trafficking and Secretion to the Parasitophorous Vacuole (PV)

### 4.1. Dense Granules and Their Role in the PV Formation

A major conserved function of the PV in coccidian parasites is to optimize growth and nutrition while avoiding host defenses [83]. However, there are several lines of evidence supporting that apicomplexan PVs display an evolutionary adaptation, with differences in the composition of the PV components of *Toxoplasma* and *Plasmodium* spp. described over a decade ago [84]. Dense granules are distinct secretory organelles described in cyst-forming coccidians and some other apicomplexans, whose contents (GRAs) are constitutively secreted into the PV during endogenous parasite replication. GRAs have crucial roles in the formation of the intravacuolar network (IVN) [85] and parasite interaction with the host cell [86,87], with some GRAs crossing the PV membrane to reach the host cytoplasm or nucleus [88]. Recent investigations have proved that Rab11A directs the transport of proteins to dense granules and the secretion of GRA proteins into the vacuolar space in *T. gondii* [49]. The N-terminal domain of TgGRA6 seems critical for its ability to target dense granules [89]. However, no specific sorting signals have yet been defined for proteins destined to this organelle. Dense granules have been defined as a default secretory pathway for soluble proteins containing a signal peptide but not for other targeting signals [26,90].

Nevertheless, the physical presence of dense granule organelles is elusive in *Eimeria* sporozoites [7]. Dense granules, as defined in *T. gondii* and other apicomplexans, have not been visualized by electron microscopy of sporozoites of *E. tenella* [7,91]. Along with a lack of visual evidence of dense granules, there is sparse conservation of genes encoding GRA protein orthologues in *Eimeria* genomes, as evidenced by the low numbers of *T. gondii* GRA orthologues found in transcriptomic [92], proteomic (in *E. tenella*) [93,94], and protein characterization studies [95], where less than 10 proteins have been identified as potential *Eimeria* GRAs in comparison to the 53 proteins annotated for *T. gondii* (www.toxodb.org, accessed on 26 August 2021). Different authors have noted the downregulation of GRA genes in the intestinal stages (merozoites) of *T. gondii* compared to tachyzoites and bradyzoites [11,96], with 12 of them absent in merozoites. Other GRA genes, such as TgGRA 2, 4, and 6, were >8-fold lower in *T. gondii* than in tachyzoites. This supports the previous finding of Ferguson and collaborators [97], which showed that TgGRA proteins 1 to 6 were not detected by specific antibodies in merozoites, while TgNTPase and TgGRA7 were expressed in merozoites [98]. These results suggest the possibility that dense granules are of biological importance, primarily in the acute and tissue cyst stages of *T. gondiii* and not in the gut stages, which correlates well with observations in the *Eimeria* species that they develop only in the intestine. Interestingly, TgGRA11 transcripts were eight-fold higher in the *Toxoplasma* gut stages [96], and their trafficking into the PV has been proved by immunofluorescence with specific antibodies and by transgenic expression in tachyzoites engineer to express this protein [99]. In a recent transcriptomic study comparing unsporulated oocysts and sporozoites of *E. necatrix*, EnGRA11 was identified. However, no differences were observed between different stages, whereas two other potential dense granules proteins, EnGRA10 and EnDG32, showed up- and downregulation in sporozoites [92]. Recent work analyzing host and parasite proteomes during endogenous development of *E. tenella* detected the presence of two GRA orthologues by mass spectrometry that were significantly upregulated after 24 hours of infection [94]. These GRA orthologues were not identified in proteomes from whole extracellular sporozoites [93] or rhoptries isolated from extracellular sporozoites [41], strongly suggesting that they are genuinely associated with endogenous development and, potentially, with PV modifications.

The fusion of an N-terminal signal peptide (SP2, from EtMIC2) to the fluorescent reporter mCherry translocates the reporter into the endomembrane system of *E. tenella* [28]. Then, the peptide traffics through the constitutive secretory pathway in a manner similar to that described for the signal-peptide-tagged reporters in *T. gondii* [26]. Freshly purified sporozoites secrete SP2-mCherry, and this is detected in the culture supernatants, as the intracellular detection of mCherry fades over time [28]. However, once the sporozoites invade host cells and become intracellular, this process appears to be under tighter regulation. mCherry is not detected within the PV as described in *T. gondii* [26], suggesting that *E. tenella* sporozoites exert specific control over what is secreted at this point of the lifecycle in contrast to *T. gondii.* This could be related to the differences in the PV structure required to support the different rates of parasite endogenous division and/or the PV lifespan (Figure 1A,C). So far, the nature of the vesicles/storage organelles transporting and permitting secretion of SP2-mCherry in *E. tenella* remains to be determined.

The analysis of the intracellular parasites of *E. tenella* by electron microscopy shows close proximity between the sporozoite pellicle and the PV membrane (PVM), which is maintained as the sporozoite rounds up to a trophozoite and early schizont ([94], Figure 1A). As new daughter merozoites form in the rounded schizont within the parasite outer membrane (from which they eventually bud from) (Figure 1A), it is feasible that proteins with a role in forming the IVN within the PV (equivalent to GRAs in *T. gondii*) remain within this parasite outer membrane. Thus, these proteins are not secreted into the reduced space between the pellicle and PVM. Thus hypothesis could explain, at least in part, the differential regulation of secretion into the PV by *E. tenella* and *T. gondii* described above [28].

### 4.2. Mysterious Trafficking to and Distribution of Refractile Bodies (RB)

The shift from an extracellular to intracellular environment can lead to significant changes in the distribution of organelles within a parasite. For example, the well-described single mitochondrion of intracellular *T. gondii* tachyzoites changes rapidly when it becomes extracellular [100]. A very noticeable change that occurs early after the *E. tenella* sporozoite invasion of host cells is a reduction in the parasite refractile bodies (RB) from two to one [101]. This phenomenon is observable by light microscopy in the cell culture due to the large size of these organelles. RB are large, non-membrane-bound inclusions of unknown function found in the sporozoites of *Eimeria* and *Lankesterella*, but not in other Eimeriidae (*Cyclospora* or *Caryspora*) or Sarcocystidae, such as *T. gondii.* Different mechanisms, such as fragmentation of the anterior RB or fusion, have been hypothesized as the cause of RB reduction [101,102,103]. However, little is known about how the parasite controls the repositioning of organelles. A recent study described the role of F-actin and an unconventional myosin motor (TgMyoF) in the dynamics and organization of the endomembrane system in *T. gondii* [104]. Nevertheless, the involvement of the action/myosin motors leading RB has not been studied in *Eimeria* spp.

A striking feature of *Eimeria* RB is that they lack a limiting membrane and have no discernible internal structures that can be visualized when chemically fixed and viewed by electron microscopy [103]. Other cytoplasmic inclusions have been described in some other coccidia, including *Cystoisospora*, as well as in more distantly related Apicomplexa, such as *Cryptosporidium* and *Plasmodium.* Interestingly, the well-characterized antigen SO7 [105] and the aspartyl proteinase eimepsin [106] are relocated from the RB to the apical end of the sporozoite during invasion [106,107]. In the coming years, the scientific community may aim to understand how the proteins stored in the RB [108] are trafficked to these membrane-less RBs—and, from there, to the apical end of the sporozoite.

## 5. Conclusions

After reviewing the studies related to protein trafficking in *T. gondii* and *Eimeria spp*., we considered these studies in the context of the described events that occur during the host-cell invasion and subsequent parasite endogenous development (Figure 1). We conclude that the mechanisms by which proteins are targeted and trafficked to the apical secretory organelles and secreted before and during invasion are broadly conserved. However, there is some divergence when it comes to intracellular parasite development and the formation of the PV, which most likely reflects the differences in the specific biological functions of the vacuoles based on their respective lifecycles. The main conclusions are summarized in Table 2.

During the past few years, important advances have been made in the knowledge of the molecular mechanisms that drive protein trafficking in *T. gondii*. This parasite has emerged as a model organism because of its amenability to be cultured in cell lines and the huge development of molecular tools, including CRISPR/Cas9, for the generation of genetic modifications, enabling studies in gene function [109,110]. By contrast, the inability of *Eimeria* sporozoites to grow productively in cell culture, difficulties in obtaining clonal lines, and the lack of molecular tools for the generation of knockouts or regulation of gene expression significantly limits mechanistic studies. Retrieving and using information from *Toxoplasma* is extremely valuable to formulate novel hypotheses about *Eimeria* biology and to develop improved strategies for the control of coccidiosis. However, it is essential that specific studies are performed directly in *Eimeria* (or in other target genera) to validate hypotheses and to understand the divergent biological mechanisms. For example, with a lack of advanced molecular technologies, high-resolution microscopy is emerging as a valuable means to study the *Eimeria* species and other coccidians [94].

Further studies on the early stages of the endogenous development of *Eimeria* species are critical, since this part of the lifecycle is required in vivo for the induction of a protective immune response [111,112,113]. This is an area of research that must be developed independently for *Eimeria* and which cannot rely solely on the data generated in *T. gondii*. The presence of an IVN and its composition during schizont development, how the IVN formed if dense granules are not present, and the interaction of parasite components with host cells are currently unknown for monoxenous Coccidia, including the *Eimeria* species. In addition, the role of the unique RB, which occupy a significant proportion of the volume of the sporozoite and early intracellular schizont, remains enigmatic. These should be areas of active research in the following years to find new strategies to target specific control measures against these parasites.

## Figures and Tables

**Table 1:** *Eimeria* spp. orthologues to the relevant molecules related to trafficking control in *T. gondii*.

Protein Name	*T. gondii* Accession No.	*Eimeria* spp. Orthologues Accession No.	Function	Reference
Rab5 (vsp21)	TGME49_267810	EAH_00052370 ETH_00021330	GTPase early ELC—Trafficking regulator	[47]
Rab7	TGME49_248880	EAH_00041220 EBH_0005250 EMWEY_00037980 ENH_00004210 EPH_0002950 ETH_00033345	GTPase late ELC—Trafficking regulator	[48]
Ras-related protein Rab11	TGME49_289680	EAH_00064080 EBH_0019080 EMWEY_00044890 EMH_0029380 ENH_00049500 EPH_0069930 ETH_00017580	GTPase—Trafficking regulator of dense granules	[49]
Vacuolar sorting protein 9 (vps9) domain-containing protein	TGME49_230140	EAH_00040570 EBH_0050290 ENH_00084230	Rab5 activator	[50]
SNARE protein	TGME49_215420	EBH_0073150 ENH_00068780 ETH_00015550	Vesicle fusion	[51,52]
Syntaxin 6, n-terminal protein	TGME49_300240	EAH_00027860 EBH_0001930 EMWEY_00046820 ENH_00049060 ETH_00005800	Vesicle fusion (SNARE) (TGN)	[53]
Dynamin-related protein DRPB (vsp1)	TGME49_321620	EBH_0054600 EMWEY_00015630 ENH_00048570 EPH_0003250 ETH_00019655	Biogenesis of secretory organelles	[54]
Clathrin heavy chain, putative	TGME49_290950	EAH_00001570 EBH_0021940 EMWEY_00028900 EMH_0048570 ENH_00082390 EPH_0001910 ETH_00021065	Biogenesis of secretory organelles	[55]
Sortilin, putative (vsp10)	TGME49_290160	EAH_00066610 EMH_0073680 ENH_00014690 ETH_00034385	Biogenesis of secretory organelles	[56]
Mu1 adaptin	TGME49_289770	EAH_00014090 EBH_0015120 EMWEY_00033330 EMH_0026560 ENH_00056570 EPH_0075080 ETH_00005150	Clathrin adaptor complex (AP1)	[49]

EAH: *E. acervulina* Houghton; EBH: *E. brunetti* Houghton; EMH: *E. mitis* Houghton; EMWEY: *E. maxima* Weybridge; ENH: *E. necatrix* Houghton; EPH: *E. praecox* Houghton; ETH: *E. tenella* Houghton.

**Table 2:** Summary of the main similarities and differences in protein trafficking and secretion between *Eimeria* sporozoites and *T. gondii* tachyzoites.

Similarities	Divergences
Early Interactions	
▪ GPI-anchors of SAG proteins contain targeting signals for external localization	▪ Specific clade of *Eimeria* ROPK
▪ Equivalent role of MICs and RON/ROPs in invasion	
▪ Similar timing of secretion of MICs	
▪ Presence of MICs and RONs orthologues	
▪ Presence of MJ structure	
Essential molecules and signals for ER/TGN/ELC trafficking	
▪ Orthologues molecules for TGN to ELC trafficking	
▪ Interchangeable signals for MIC/ROPs trafficking	
▪ Potential distinct populations of micronemes	
Regulation of MIC/ROPs secretion	
▪ MICs secreted prior to ROPs	
▪ ROPs reach the host cell cytoplasm/nucleus	
Dense granules and PV formation	
▪ *E. tenella* GRAs overexpressed during endogenous development	▪ Lack of visual evidence of dense granules
	▪ Low GRA orthologues
	▪ Down regulation of GRAs in *T. gondii* gut stages
	▪ Differential control of secretion into PV
Trafficking to RB	
	▪ Unique presence in *Eimeria* and *Lankesterella*
	▪ Trafficking to/from membrane-less organelle

## Data Availability

Not applicable.

## References

[1] Lingelbach K., Joiner K.A. (1998). The parasitophorous vacuole membrane surrounding Plasmodium and Toxoplasma: An unusual compartment in infected cells. J. Cell Sci..

[2] Sangaré L.O., Alayi T.D., Westermann B., Hovasse A., Sindikubwabo F., Callebaut I., Werkmeister E., Lafont F., Slomianny C., Hakimi M.-A. (2016). Unconventional endosome-like compartment and retromer complex in Toxoplasma gondii govern parasite integrity and host infection. Nat. Commun..

[3] Ngô H.M., Yang M., Paprotka K., Pypaert M., Hoppe H., Joiner K. (2003). AP-1 in Toxoplasma gondii Mediates Biogenesis of the Rhoptry Secretory Organelle from a Post-Golgi Compartment. J. Biol. Chem..

[4] Tomavo S., Slomianny C., Meissner M., Carruthers V.B. (2013). Protein Trafficking through the Endosomal System Prepares Intracellular Parasites for a Home Invasion. PLoS Pathog..

[5] Tenter A.M., Barta J.R., Beveridge I., Duszynski D.W., Mehlhorn H., Morrison D.A., Thompson R.C.A., Conrad P.A. (2002). The conceptual basis for a new classification of the coccidia. Int. J. Parasitol..

[6] Blake D.P., Knox J., Dehaeck B., Huntington B., Rathinam T., Ravipati V., Ayoade S., Gilbert W., Adebambo A.O., Jatau I.D. (2020). Re-calculating the cost of coccidiosis in chickens. Veter- Res..

[7] Burrell A., Tomley F.M., Vaughan S., Marugan-Hernandez V. (2019). Life cycle stages, specific organelles and invasion mechanisms of Eimeria species. Parasitology.

[8] Ferguson D.J.P., Sahoo N., Pinches R.A., Bumstead J.M., Tomley F.M., Gubbels M.-J. (2008). MORN1 Has a Conserved Role in Asexual and Sexual Development across the Apicomplexa. Eukaryot. Cell.

[9] Di Cristina M., Marocco D., Galizi R., Proietti C., Spaccapelo R., Crisanti A. (2008). Temporal and spatial distribution of Toxoplasma gondii differentiation into Bradyzoites and tissue cyst formation in vivo. Infect. Immun..

[10] Bohne W., Heesemann J., Gross U. (1994). Reduced replication of Toxoplasma gondii is necessary for induction of bradyzoite-specific antigens: A possible role for nitric oxide in triggering stage conversion. Infect. Immun..

[11] Behnke M.S., Zhang T.P., Dubey J.P., Sibley L.D. (2014). Toxoplasma gondii merozoite gene expression analysis with comparison to the life cycle discloses a unique expression state during enteric development. BMC Genom..

[12] Koreny L., Zeeshan M., Barylyuk K., Tromer E.C., van Hooff J.J.E., Brady D., Ke H., Chelaghma S., Ferguson D.J.P., Eme L. (2021). Molecular characterization of the conoid complex in Toxoplasma reveals its conservation in all apicomplexans, including Plasmodium species. PLoS Biol..

[13] Pacheco N.D., Vetterling J.M., Doran D.J. (1975). Ultrastructure of Cytoplasmic and Nuclear Changes in Eimeria tenella during First-Generation Schizogony in Cell Culture. J. Parasitol..

[14] Ferguson D., Hutchison W.M., Dunachie J.F., Siim J.C. (2009). Ultrastructural study of early stages of asexual multiplication and microgametogony of toxoplasma gondii in the small intestine of the cat. Acta Pathol. Microbiol. Scand. Sect. B Microbiol. Immunol..

[15] Dubey J.P., Lindsay D.S., Speer C.A. (1998). Structures of Toxoplasma gondii Tachyzoites, Bradyzoites, and Sporozoites and Biology and Development of Tissue Cysts. Clin. Microbiol. Rev..

[16] Aikawa M., Komata Y., Asai T., Midorikawa O. (1977). Transmission and scanning electron microscopy of host cell entry by Toxoplasma gondii. Am. J. Pathol..

[17] Bumstead J., Tomley F. (2000). Induction of secretion and surface capping of microneme proteins in Eimeria tenella. Mol. Biochem. Parasitol..

[18] Besteiro S., Dubremetz J.-F., Lebrun M. (2011). The moving junction of apicomplexan parasites: A key structure for invasion. Cell. Microbiol..

[19] Jenkins M.C., Dame J.B. (1987). Identification of immunodominant surface antigens of Eimeria acervulina sporozoites and merozoites. Mol. Biochem. Parasitol..

[20] Grimwood J., Smith J. (1992). Toxoplasma gondii: The role of a 30-kDa surface protein in host cell invasion. Exp. Parasitol..

[21] Kim S.K., Boothroyd J.C. (2005). Stage-specific expression of surface antigens by Toxoplasma gondii as a mechanism to facilitate parasite persistence. J. Immunol..

[22] Tabares E., Ferguson D., Clark J., Soon P.E., Wan K.L., Tomley F. (2004). Eimeria tenella sporozoites and merozoites differentially express glycosylphosphatidylinositol-anchored variant surface proteins. Mol. Biochem. Parasitol..

[23] Reid A., Blake D., Ansari H.R., Billington K., Browne H., Bryant J., Dunn M., Hung S.S., Kawahara F., Miranda-Saavedra D. (2014). Genomic analysis of the causative agents of coccidiosis in domestic chickens. Genome Res..

[24] Ramly N.Z., Dix S.R., Ruzheinikov S.N., Sedelnikova S.E., Baker P.J., Chow Y.P., Tomley F.M., Blake D.P., Wan K.-L., Nathan S. (2021). The structure of a major surface antigen SAG19 from Eimeria tenella unifies the Eimeria SAG family. Commun. Biol..

[25] Crawford J., Lamb E., Wasmuth J., Grujic O., Grigg M.E., Boulanger M.J. (2010). Structural and functional characterization of SporoSAG: A SAG2-related surface antigen from Toxoplasma gondii. J. Biol. Chem..

[26] Karsten V., Qi H., Beckers C.J., Reddy A., Dubremetz J.-F., Webster P., Joiner K. (1998). The Protozoan Parasite Toxoplasma gondii Targets Proteins to Dense Granules and the Vacuolar Space Using Both Conserved and Unusual Mechanisms. J. Cell Biol..

[27] Reiss M., Viebig N., Brecht S., Fourmaux M.-N., Soete M., Di Cristina M., Dubremetz J.F., Soldati D. (2001). Identification and Characterization of an Escorter for Two Secretory Adhesins in Toxoplasma gondii. J. Cell Biol..

[28] Marugan-Hernandez V., Long E., Blake D., Crouch C., Tomley F. (2017). Eimeria tenella protein trafficking: Differential regulation of secretion versus surface tethering during the life cycle. Sci. Rep..

[29] Miller S.A., Binder E.M., Blackman M.J., Carruthers V., Kim K. (2001). A Conserved Subtilisin-like Protein TgSUB1 in Microneme Organelles of Toxoplasma gondii. J. Biol. Chem..

[30] De Souza W., Souto-Padron T. (1978). Ultrastructural localization of basic proteins on the conoid, rhoptries and micronemes of Toxoplasma gondii. Z Parasitenkd..

[31] Hu K., Roos D., Murray J.M. (2002). A novel polymer of tubulin forms the conoid of Toxoplasma gondii. J. Cell Biol..

[32] Ryan R., Shirley M., Tomley F. (2000). Mapping and expression of microneme genes in Eimeria tenella. Int. J. Parasitol..

[33] Brown P.J., Billington K.J., Bumstead J.M., Clark J.D., Tomley F.M. (2000). A microneme protein from Eimeria tenella with homology to the Apple domains of coagulation factor XI and plasma pre-kallikrein. Mol. Biochem. Parasitol..

[34] Rabenau K.E., Sohrabi A., Tripathy A., Reitter C., Ajioka J.W., Tomley F.M., Carruthers V.B. (2001). TgM2AP participates in Toxoplasma gondii invasion of host cells and is tightly associated with the adhesive protein TgMIC2. Mol. Microbiol..

[35] Periz J., Gill A., Hunt L., Brown P., Tomley F.M. (2007). The Microneme Proteins EtMIC4 and EtMIC5 of Eimeria tenella Form a Novel, Ultra-high Molecular Mass Protein Complex That Binds Target Host Cells. J. Biol. Chem..

[36] Lai L., Bumstead J., Liu Y., Garnett J., Campanero-Rhodes M.A., Blake D., Palma A.D.S., Chai W., Ferguson D., Simpson P. (2011). The Role of Sialyl Glycan Recognition in Host Tissue Tropism of the Avian Parasite Eimeria tenella. PLoS Pathog..

[37] Clough B., Wright J.D., Pereira P.M., Hirst E.M., Johnston A.C., Henriques R., Frickel E.M. (2016). K63-Linked Ubiquitination Targets Toxoplasma gondii for Endo-lysosomal Destruction in IFNgamma-Stimulated Human Cells. PLoS Pathog..

[38] Saeij J., Coller S., Boyle J.P., Jerome M.E., White M.W., Boothroyd J.C. (2006). Toxoplasma co-opts host gene expression by injection of a polymorphic kinase homologue. Nature.

[39] Wang J.-L., Li T.-T., Elsheikha H., Chen K., Zhu W.-N., Yue D.-M., Zhu X.-Q., Huang S.-Y. (2017). Functional Characterization of Rhoptry Kinome in the Virulent Toxoplasma gondii RH Strain. Front. Microbiol..

[40] Carruthers V.B., Tomley F.M. (2008). Microneme Proteins in Apicomplexans. Mol. Mech. Parasite Invasion.

[41] Oakes R.D., Kurian D., Bromley E., Ward C., Lal K., Blake D.P., Reid A.J., Pain A., Sinden R.E., Wastling J.M. (2012). The rhoptry proteome of Eimeria tenella sporozoites. Int. J. Parasitol..

[42] Diallo M.A., Sausset A., Gnahoui-David A., Silva A.R.E., Brionne A., Le Vern Y., Bussière F.I., Tottey J., Lacroix-Lamandé S., Laurent F. (2019). Eimeria tenella ROP kinase EtROP1 induces G0/G1 cell cycle arrest and inhibits host cell apoptosis. Cell. Microbiol..

[43] Talevich E., Kannan N. (2013). Structural and evolutionary adaptation of rhoptry kinases and pseudokinases, a family of coccidian virulence factors. BMC Evol. Biol..

[44] Nishi M., Hu K., Murray J.M., Roos D.S. (2008). Organellar dynamics during the cell cycle of Toxoplasma gondii. J. Cell Sci..

[45] McGovern O.L., Rivera-Cuevas Y., Kannan G., Narwold A.J., Carruthers V.B. (2018). Intersection of endocytic and exocytic systems inToxoplasma gondii. Traffic.

[46] Dogga S.K., Mukherjee B., Jacot D., Kockmann T., Molino L., Hammoudi P.-M., Hartkoorn R.C., Hehl A.B., Soldati-Favre D. (2017). A druggable secretory protein maturase of Toxoplasma essential for invasion and egress. eLife.

[47] Robibaro B., Stedman T.T., Coppens I., Ngo H.M., Pypaert M., Bivona T., Nam H.W., Joiner K.A. (2002). Toxoplasma gondii Rab5 enhances cholesterol acquisition from host cells. Cell. Microbiol..

[48] Tomavo S. (2014). Evolutionary repurposing of endosomal systems for apical organelle biogenesis in Toxoplasma gondii. Int. J. Parasitol..

[49] Venugopal K., Chehade S., Werkmeister E., Barois N., Periz J., Lafont F., Tardieux I., Khalife J., Langsley G., Meissner M. (2020). Rab11A regulates dense granule transport and secretion during Toxoplasma gondii invasion of host cells and parasite replication. PLoS Pathog..

[50] Sakura T., Sindikubwabo P.F., Oesterlin L.K., Bousquet H., Slomianny C., Hakimi M.-A., Langsley G., Tomavo S. (2016). A Critical Role for Toxoplasma gondii Vacuolar Protein Sorting VPS9 in Secretory Organelle Biogenesis and Host Infection. Sci. Rep..

[51] Chaturvedi S., Qi H., Coleman D., Rodriguez A., Hanson P.I., Striepen B., Roos D., Joiner K.A. (1999). Constitutive Calcium-independent Release of Toxoplasma gondii Dense Granules Occurs through the NSF/SNAP/SNARE/Rab Machinery. J. Biol. Chem..

[52] Bisio H., Ben Chaabene R., Sabitzki R., Maco B., Marq J.B., Gilberger T.-W., Spielmann T., Soldati-Favre D. (2020). The ZIP Code of Vesicle Trafficking in Apicomplexa: SEC1/Munc18 and SNARE Proteins. mBio.

[53] Jackson A.J., Clucas C., Mamczur N.J., Ferguson D.J., Meissner M. (2013). Toxoplasma gondiiSyntaxin 6 Is Required for Vesicular Transport Between Endosomal-Like Compartments and the Golgi Complex. Traffic.

[54] Breinich M.S., Ferguson D., Foth B.J., van Dooren G., Lebrun M., Quon D.V., Striepen B., Bradley P.J., Frischknecht F., Carruthers V. (2009). A Dynamin Is Required for the Biogenesis of Secretory Organelles in Toxoplasma gondii. Curr. Biol..

[55] Pieperhoff M.S., Schmitt M., Ferguson D.J.P., Meissner M. (2013). The Role of Clathrin in Post-Golgi Trafficking in Toxoplasma gondii. PLoS ONE.

[56] Sloves P.-J., Delhaye S., Mouveaux T., Werkmeister E., Slomianny C., Hovasse A., Alayi T.D., Callebaut I., Gaji R.Y., Schaeffer-Reiss C. (2012). Toxoplasma Sortilin-like Receptor Regulates Protein Transport and Is Essential for Apical Secretory Organelle Biogenesis and Host Infection. Cell Host Microbe.

[57] Harper J.M., Huynh M.-H., Coppens I., Parussini F., Moreno S., Carruthers V.B. (2006). A Cleavable Propeptide InfluencesToxoplasmaInfection by Facilitating the Trafficking and Secretion of the TgMIC2–M2AP Invasion Complex. Mol. Biol. Cell.

[58] El Hajj H., Papoin J., Cerede O., Garcia-Reguet N., Soete M., Dubremetz J.F., Lebrun M. (2008). Molecular signals in the trafficking of Toxoplasma gondii protein MIC3 to the micronemes. Eukaryot. Cell.

[59] Brydges S.D., Zhou X.W., Huynh M.-H., Harper J.M., Mital J., Adjogble K.D.Z., Däubener W., Ward G.E., Carruthers V. (2006). Targeted Deletion of MIC5 Enhances Trimming Proteolysis of Toxoplasma Invasion Proteins. Eukaryot. Cell.

[60] Bradley P.J., Boothroyd J.C. (2001). The pro region of Toxoplasma ROP1 is a rhoptry-targeting signal. Int. J. Parasitol..

[61] Binder E.M., Lagal V., Kim K. (2008). The Prodomain ofToxoplasma gondiiGPI-Anchored Subtilase TgSUB1 Mediates its Targeting to Micronemes. Traffic.

[62] Di Cristina M., Spaccapelo R., Soldati D., Bistoni F., Crisanti A. (2000). Two conserved amino acid motifs mediate protein targeting to the micronemes of the apicomplexan parasite Toxoplasma gondii. Mol. Cell. Biol..

[63] Hoppe H., Ngô H.M., Yang M., Joiner K. (2000). Targeting to rhoptry organelles of Toxoplasma gondii involves evolutionarily conserved mechanisms. Nature.

[64] Marks M., Ohno H., Kirchnausen T., Bonifacino J.S. (1997). Protein sorting by tyrosine-based signals: Adapting to the Ys and wherefores. Trends Cell Biol..

[65] Gaji R.Y., Flammer H.P., Carruthers V. (2011). Forward Targeting of Toxoplasma gondii Proproteins to the Micronemes Involves Conserved Aliphatic Amino Acids. Traffic.

[66] Huynh M.-H., Opitz C., Kwok L.-Y., Tomley F.M., Carruthers V., Soldati-Favre D. (2004). Trans-genera reconstitution and complementation of an adhesion complex in Toxoplasma gondii. Cell. Microbiol..

[67] Kremer K., Kamin D., Rittweger E., Wilkes J., Flammer H., Mahler S., Heng J., Tonkin C.J., Langsley G., Hell S.W. (2013). An Overexpression Screen of Toxoplasma gondii Rab-GTPases Reveals Distinct Transport Routes to the Micronemes. PLoS Pathog..

[68] Pastor-Fernández I., Kim S., Billington K., Bumstead J., Marugan-Hernandez V., Küster T., Ferguson D.J., Vervelde L., Blake D., Tomley F.M. (2018). Development of cross-protective Eimeria-vectored vaccines based on apical membrane antigens. Int. J. Parasitol..

[69] Portes J., Barrias E., Travassos R., Attias M., De Souza W. (2020). Toxoplasma gondii Mechanisms of Entry Into Host Cells. Front. Cell. Infect. Microbiol..

[70] Paredes-Santos T.C., de Souza W., Attias M. (2011). Dynamics and 3D organization of secretory organelles of Toxoplasma gondii. J. Struct. Biol..

[71] Frénal K., Dubremetz J.-F., Lebrun M., Soldati-Favre D. (2017). Gliding motility powers invasion and egress in Apicomplexa. Nat. Rev. Genet..

[72] Lovett J.L., Marchesini N., Moreno S.N., Sibley L.D. (2002). Toxoplasma gondii microneme secretion involves intracellular Ca(^2+^) release from inositol 1,4,5-triphosphate (IP(3))/ryanodine-sensitive stores. J. Biol. Chem..

[73] Bullen H.E., Bisio H., Soldati-Favre D. (2019). The triumvirate of signaling molecules controlling Toxoplasma microneme exocytosis: Cyclic GMP, calcium, and phosphatidic acid. PLoS Pathog..

[74] Dubois D.J., Soldati-Favre D. (2019). Biogenesis and secretion of micronemes inToxoplasma gondii. Cell. Microbiol..

[75] Ihara F., Nishikawa Y. (2021). Toxoplasma gondii manipulates host cell signaling pathways via its secreted effector molecules. Parasitol. Int..

[76] Kessler H., Herm-Götz A., Hegge S., Rauch M., Soldati-Favre D., Frischknecht F., Meissner M. (2008). Microneme protein 8—A new essential invasion factor in Toxoplasma gondii. J. Cell Sci..

[77] Aquilini E., Cova M.M., Mageswaran S.K., Pacheco N.D.S., Sparvoli D., Penarete-Vargas D.M., Najm R., Graindorge A., Suarez C., Maynadier M. (2021). An Alveolata secretory machinery adapted to parasite host cell invasion. Nat. Microbiol..

[78] Suarez C., Lentini G., Ramaswamy R., Maynadier M., Aquilini E., Berry-Sterkers L., Cipriano M., Chen A.L., Bradley P., Striepen B. (2019). A lipid-binding protein mediates rhoptry discharge and invasion in Plasmodium falciparum and Toxoplasma gondii parasites. Nat. Commun..

[79] Coleman B.I., Saha S., Sato S., Engelberg K., Ferguson D.J.P., Coppens I., Lodoen M.B., Gubbels M.-J. (2018). A Member of the Ferlin Calcium Sensor Family Is Essential for Toxoplasma gondii Rhoptry Secretion. mBio.

[80] Håkansson S., Charron A.J., Sibley L. (2001). Toxoplasma evacuoles: A two-step process of secretion and fusion forms the parasitophorous vacuole. EMBO J..

[81] Nichols B.A., Chiappino M.L., O’Connor G.R. (1983). Secretion from the Rhoptries of Toxoplasma gondii during host-cell invasion. J. Ultrastruct. Res..

[82] Sparvoli D., Lebrun M. (2021). Unraveling the Elusive Rhoptry Exocytic Mechanism of Apicomplexa. Trends Parasitol..

[83] Lemgruber L., De Souza W., Vommaro R.C. (2008). Freeze-fracture study of the dynamics of Toxoplasma gondii parasitophorous vacuole development. Micron.

[84] Mercier C., Adjogble K.D., Däubener W., Cesbron-Delauw M.-F. (2005). Dense granules: Are they key organelles to help understand the parasitophorous vacuole of all Apicomplexa parasites?. Int. J. Parasitol..

[85] Sibley L., Niesman I., Parmley S., Cesbron-Delauw M. (1995). Regulated secretion of multi-lamellar vesicles leads to formation of a tubulo-vesicular network in host-cell vacuoles occupied by Toxoplasma gondii. J. Cell Sci..

[86] Dou Z., McGovern O.L., Di Cristina M., Carruthers V.B. (2014). Toxoplasma gondii Ingests and Digests Host Cytosolic Proteins. mBio.

[87] Gold D.A., Kaplan A.D., Lis A., Bett G., Rosowski E., Cirelli K.M., Bougdour A., Sidik S.M., Beck J.R., Lourido S. (2015). The Toxoplasma Dense Granule Proteins GRA17 and GRA23 Mediate the Movement of Small Molecules between the Host and the Parasitophorous Vacuole. Cell Host Microbe.

[88] Bougdour A., Durandau E., Brenier-Pinchart M.-P., Ortet P., Barakat M., Kieffer S., Curt-Varesano A., Curt-Bertini R.-L., Bastien O., Couté Y. (2013). Host Cell Subversion by Toxoplasma GRA16, an Exported Dense Granule Protein that Targets the Host Cell Nucleus and Alters Gene Expression. Cell Host Microbe.

[89] Labruyere E., Lingnau M., Mercier C., Sibley L. (1999). Differential membrane targeting of the secretory proteins GRA4 and GRA6 within the parasitophorous vacuole formed by Toxoplasma gondii. Mol. Biochem. Parasitol..

[90] Sheiner L., Soldati-Favre D., Soldati-Favre M. (2008). Protein Trafficking inside Toxoplasma gondii. Traffic.

[91] Danforth H.D., Augustine P.C., Clare A.R. (1994). Ultrastructural observations of development of Eimeria tenella in a novel established avian-derived cell line. Parasitol. Res..

[92] Gao Y., Suding Z., Wang L., Liu D., Su S., Xu J., Hu J., Tao J. (2021). Full-length transcriptome sequence analysis of Eimeria necatrix unsporulated oocysts and sporozoites identifies genes involved in cellular invasion. Veter- Parasitol..

[93] Lal K., Bromley E., Oakes R., Prieto J.H., Sanderson S.J., Kurian D., Hunt L., Yates J.R., Wastling J.M., Sinden R.E. (2009). Proteomic comparison of four Eimeria tenella life-cycle stages: Unsporulated oocyst, sporulated oocyst, sporozoite and second-generation merozoite. Proteomics.

[94] Marugan-Hernandez V., Jeremiah G., Aguiar-Martins K., Burrell A., Vaughan S., Xia D., Randle N., Tomley F. (2020). The Growth of Eimeria tenella: Characterization and Application of Quantitative Methods to Assess Sporozoite Invasion and Endogenous Development in Cell Culture. Front. Cell. Infect. Microbiol..

[95] Kong P., Lehmann M.J., Helms J.B., Brouwers J.F., Gupta N. (2018). Lipid analysis of Eimeria sporozoites reveals exclusive phospholipids, a phylogenetic mosaic of endogenous synthesis, and a host-independent lifestyle. Cell Discov..

[96] Hehl A.B., Basso W.U., Lippuner C., Ramakrishnan C., Okoniewski M., Walker A.R., Grigg E.M., Smith N.C., Deplazes P. (2015). Asexual expansion of Toxoplasma gondii merozoites is distinct from tachyzoites and entails expression of non-overlapping gene families to attach, invade, and replicate within feline enterocytes. BMC Genom..

[97] Ferguson D.J., Cesbron-Delauw M.F., Dubremetz J.F., Sibley L.D., Joiner K.A., Wright S. (1999). The expression and distribution of dense granule proteins in the enteric (Coccidian) forms of Toxoplasma gondii in the small intestine of the cat. Exp. Parasitol..

[98] Ferguson D.J.P., Jacobs D., Saman E., Dubremetz J.-F., Wright S.E. (1999). In vivo expression and distribution of dense granule protein 7 (GRA7) in the exoenteric (tachyzoite, bradyzoite) and enteric (coccidian) forms of Toxoplasma gondii. Parasitology.

[99] Ramakrishnan C., Walker R.A., Eichenberger R.M., Hehl A., Smith N. (2017). The merozoite-specific protein, TgGRA11B, identified as a component of the Toxoplasma gondii parasitophorous vacuole in a tachyzoite expression model. Int. J. Parasitol..

[100] Ovciarikova J., Lemgruber L., Stilger K.L., Sullivan W.J., Sheiner L. (2017). Mitochondrial behaviour throughout the lytic cycle of Toxoplasma gondii. Sci. Rep..

[101] Fayer R., Hammond D.M. (1969). Morphological Changes in Eimeria bovis Sporozoites during Their First Day in Cultured Mammalian Cells. J. Parasitol..

[102] Clark W.N., Hammond D.M. (1969). Development of Eimeria auburnensis in Cell Cultures. J. Protozool..

[103] Roberts W.L., Hammond D.M. (1970). Ultrastructural and Cytologic Studies of the Sporozoites of Four Eimeria Species. J. Protozool..

[104] Carmeille R., Lomoriello P.S., Devarakonda P.M., Kellermeier J.A., Heaslip A.T. (2021). Actin and an unconventional myosin motor, TgMyoF, control the organization and dynamics of the endomembrane network in Toxoplasma gondii. PLoS Pathog..

[105] Danforth H., Augustine P. (1989). Eimeria tenella: Use of a monoclonal antibody in determining the intracellular fate of the refractile body organelles and the effect on in vitro development. Exp. Parasitol..

[106] Jean L., Grosclaude J., Labbé M., Tomley F., Péry P. (2000). Differential localisation of an Eimeria tenella aspartyl proteinase during the infection process. Int. J. Parasitol..

[107] Fetterer R.H., Jenkins M.C., Miska K.B., Barfield R.C. (2007). Characterization of the antigen so7 during development of eimeria tenella. J. Parasitol..

[108] De Venevelles P., Francois Chich J., Faigle W., Lombard B., Loew D., Péry P., Labbé M. (2006). Study of proteins associated with the Eimeria tenella refractile body by a proteomic approach. Int. J. Parasitol..

[109] Sidik S.M., Huet D., Ganesan S.M., Huynh M.-H., Wang T., Nasamu A., Thiru P., Saeij J.P., Carruthers V.B., Niles J.C. (2016). A Genome-wide CRISPR Screen in Toxoplasma Identifies Essential Apicomplexan Genes. Cell.

[110] Young J., Dominicus C., Wagener J., Butterworth S., Ye X., Kelly G., Ordan M., Saunders B., Instrell R., Howell M. (2019). A CRISPR platform for targeted in vivo screens identifies Toxoplasma gondii virulence factors in mice. Nat. Commun..

[111] McDonald V., Rose M.E., Jeffers T.K. (1986). Eimeria tenella: Immunogenicity of the first generation of schizogony. Parasitology.

[112] McDonald V., Wisher M.H., Rose E.M., Jeffers T.K. (1988). Eimeria tenella: Immunological diversity between asexual generations. Parasite Immunol..

[113] Jenkins M.C., Augustine P.C., Danforth H.D., Barta J.R. (1991). X-irradiation of Eimeria tenella oocysts provides direct evidence that sporozoite invasion and early schizont development induce a protective immune response(s). Infect. Immun..

